# Supplementary material for: A re-inducible gap gene cascade patterns the anterior–posterior axis of insects in a threshold-free fashion
Source: eLife. 2018 Dec 20;7:e41208. doi: 10.7554/eLife.41208 (PMC6329609; doi:10.7554/eLife.41208)
Supplement: Supplementary file 1. — Matlab implementations for the different models presented in our study and the parameter sets used to generate each of our simulations (Videos 1–12). [file elife-41208-supp1.docx]

**Matlab implementation of models and simulations**

Here we provide Matlab implementations for the different models presented in our study. We will then provide the parameter sets used to generated each of our simulations (Videos 1-12).

**Code C1.** Matlab code for the Module Switching realization of the Speed Regulation model

function speed_regulation_cricuit

%Module Switching realization of the Speed Regulation model.

clear

clc

close all

%%%%%%%%%%%%%%%%%%%%% PARAMETERS %%%%%%%%%%%%%%%%%%%%%%%%%%%%%%%%%%

%%% For pure short-germ: b_to_g_time=.01; a=.05; m=100;

%%% For pure long germ: b_to_g_time= 10; a=.25; m=20;

%%% For intermediate germ: b_to_g_time= 2; a=.25; m=20;

b_to_g_time=2;%time of blastoderm to germband transition

a=0.25;%starting infliction point of the gradient

m=20;%starting steepness of the gradient

v=.05;%wavefront velocity

%Gradient buildup dynamics

DyFlag=0; %gradient buildup dynamics flag:

%'0' for no buildup or decay dynamics

%'1' for only building up

%'2' for build up then decay

%'3' for only decay

%%%%%%%%%%%%%%%%%%%%%%%%%%%%%%%%%%%%%%%%%%%%%%%%%%%%%%%%%%%%%%%%%55

t=0:.01:10; %time axis

AP_axis=0:.001:.5; % spatial axis

%solve model

spacetime=zeros(5,length(t),length(AP_axis));

for pos=1:length(AP_axis)

initial_conditions=...

[.6 zeros(1,4) AP_axis(pos) b_to_g_time a v m DyFlag max(t)];

[~, x]= ode45(@odefun,t,initial_conditions);

spacetime(:,:,pos)=x(:,1:5)';

end

%plot solution

close all

for nt=1:length(t)

%setting up the gradient G

if(t(nt)<b_to_g_time)

u=0;

current_m=m;

else

u=v;

current_m=m*exp(t(nt)-b_to_g_time);

if(current_m>100)

current_m=100;

end

end

if(DyFlag==0)

Dy=1; %no buildup or decay dynamics

elseif(DyFlag==1)

Dy=1-(t(nt)/max(t)-1).^2;%only building up

elseif(DyFlag==2)

Dy=1-(2*t(nt)/max(t)-1).^2;%buildup then decay

elseif(DyFlag==3)

Dy=1-(t(nt)/max(t)).^2;%only decay

Dy(Dy<0)=0;

else

error('invalid DyFlag value')

end

G= Dy * 1.5./(1+exp(-current_m*((AP_axis-u*(t(nt)-b_to_g_time))-a)));

set(gca, 'ColorOrder',...

[86 128 193; 229 51 50; 79 185 118; 219 152 40; 168 124 78;128 128 128 ]/256,...

'NextPlot', 'replacechildren');

plot(AP_axis,[squeeze(spacetime(:,nt,:))' G'],'LineWidth',5)

axis([0 max(AP_axis) 0 2.5])

axis off

pause(.0001)

end

%%%%% Model Differential Equations %%%%%%

function dx = odefun(t,x)

%passed variables and parameters

x1=x(1);

x2=x(2);

x3=x(3);

x4=x(4);

x5=x(5);

AP_axis=x(6);%position

b_to_g_time=x(7);%blastoderm-to-germband transition time

a=x(8);%starting infliction point of the gradient

v=x(9);%velocity of the wavefront

m=x(10);%starting steepness of the gradient

DyFlag=x(11);%gradient buildup dynamics flag

max_t=x(12);%time of end of simulation

%setting up the gradient G

if(t<b_to_g_time)

u=0;

current_m=m;

else

u=v;

current_m=20*exp(t-b_to_g_time);

if(current_m>100)

current_m=100;

end

end

if(DyFlag==0)

Dy=1;

elseif(DyFlag==1)

Dy=1-(t/max_t-1).^2;

elseif(DyFlag==2)

Dy=1-(2*t/max_t-1).^2;

elseif(DyFlag==3)

Dy=1-(t/max_t).^2;

Dy(Dy<0)=0;

else

error('invalid DyFlag value')

end

G= Dy * 1.5/(1+exp(-current_m*((AP_axis-u*(t-b_to_g_time))-a)));

%gene regulation parameters

w=2.5;%dissociation constant of weak regulatory interactions

s=.4;%dissociation constant of strong regulatory interactions

n=5;%cooperativity constant

lambda=1;%decay rate

%dynamic module

dynamic(1)= G/(1+G)*1/(1+(x2/s)^n)*1/(1+(x3/s)^n)*1/(1+(x4/s)^n)*1/(1+(x5/s)^n);

dynamic(2)= G/(1+G)*1/(1+(x1/w)^n)*1/(1+(x3/s)^n)*1/(1+(x4/s)^n)*1/(1+(x5/s)^n);

dynamic(3)= G/(1+G)*1/(1+(x1/s)^n)*1/(1+(x2/w)^n)*1/(1+(x4/s)^n)*1/(1+(x5/s)^n);

dynamic(4)= G/(1+G)*1/(1+(x1/s)^n)*1/(1+(x2/s)^n)*1/(1+(x3/w)^n)*1/(1+(x5/s)^n);

dynamic(5)= G/(1+G)*1/(1+(x1/s)^n)*1/(1+(x2/s)^n)*1/(1+(x3/s)^n)*1/(1+(x4/w)^n);

%static module

static(1)= 1/(1+G)*1/(1+(x2/s)^n)*1/(1+(x3/s)^n)*1/(1+(x4/s)^n)*1/(1+(x5/s)^n);

static(2)= 1/(1+G)*1/(1+(x1/s)^n)*1/(1+(x3/s)^n)*1/(1+(x4/s)^n)*1/(1+(x5/s)^n);

static(3)= 1/(1+G)*1/(1+(x1/s)^n)*1/(1+(x2/s)^n)*1/(1+(x4/s)^n)*1/(1+(x5/s)^n);

static(4)= 1/(1+G)*1/(1+(x1/s)^n)*1/(1+(x2/s)^n)*1/(1+(x3/s)^n)*1/(1+(x5/s)^n);

static(5)= 1/(1+G)*1/(1+(x1/s)^n)*1/(1+(x2/s)^n)*1/(1+(x3/s)^n)*1/(1+(x4/s)^n);

%%%%%%%%%%%%%%%%%%%% heat-shock experiment setup %%%%%%%%%%%%%%%%%%

hs=0;

if(t>4 && t<5)

hs=0;%3

end

%%%%%%%%%%%%%%%%%%%%%%%%%%%%%%%%%%%%%%%%%%%%%%%%%%%%%%%%%%%%%%%%%%%

%total gene regulation

c=2;

d=3;

dx(1)= hs+c*static(1)+d*dynamic(1)-lambda*x1;

dx(2)= c*static(2)+d*dynamic(2)-lambda*x2;

dx(3)= c*static(3)+d*dynamic(3)-lambda*x3;

dx(4)= c*static(4)+d*dynamic(4)-lambda*x4;

dx(5)= c*static(5)+d*dynamic(5)-lambda*x5;

%zero change in parameters

dx(6:12)=0;

dx=dx';

**Code C2.** Matlab code for the French Flag GRN realization introduced in our study

function french_flag_cricuit

clear

clc

close all

%%%%%%%%%%%%%%%%%%%%% PARAMETERS %%%%%%%%%%%%%%%%%%%%%%%%%%%%%%%%%%

%%% For pure short-germ: b_to_g_time=.01; a=.05; m=100;

%%% For pure long germ: b_to_g_time= 10; a=.25; m=20;

%%% For intermediate germ: b_to_g_time= 2; a=.25; m=20;

b_to_g_time=10;%time of blastoderm to germband transition

a=0.25;%starting infliction point of the gradient

m=20;%starting steepness of the gradient

v=.05;%wavefront velocity

%Gradient buildup dynamics

DyFlag=1; %gradient buildup dynamics flag:

%'0' for no buildup or decay dynamics

%'1' for only building up

%'2' for build up then decay

%'3' for only decay

%%%%%%%%%%%%%%%%%%%%%%%%%%%%%%%%%%%%%%%%%%%%%%%%%%%%%%%%%%%%%%%%%55

t=0:.01:10; %time axis

AP_axis=0:.001:.5; % spatial axis

%solve model

spacetime=zeros(5,length(t),length(AP_axis));

for pos=1:length(AP_axis)

initial_conditions=...

[.6 zeros(1,4) AP_axis(pos) b_to_g_time a v m DyFlag max(t)];

[~, x]= ode45(@odefun,t,initial_conditions);

spacetime(:,:,pos)=x(:,1:5)';

end

%plot solution

close all

for nt=1:length(t)

%setting up the gradient G

if(t(nt)<b_to_g_time)

u=0;

current_m=m;

else

u=v;

current_m=m*exp(t(nt)-b_to_g_time);

if(current_m>100)

current_m=100;

end

end

if(DyFlag==0)

Dy=1;

elseif(DyFlag==1)

Dy=1-(t(nt)/max(t)-1).^2;

elseif(DyFlag==2)

Dy=1-(2*t(nt)/max(t)-1).^2;

elseif(DyFlag==3)

Dy=1-(t(nt)/max(t)).^2;

Dy(Dy<0)=0;

else

error('invalid DyFlag value')

end

G= Dy * 1.5./(1+exp(-current_m*((AP_axis-u*(t(nt)-b_to_g_time))-a)));

set(gca, 'ColorOrder',...

[86 128 193; 229 51 50; 79 185 118; 219 152 40; 168 124 78; 128 128 128]/256,...

'NextPlot', 'replacechildren');

plot(AP_axis,[squeeze(spacetime(:,nt,:))' G'],'LineWidth',5)

axis([0 max(AP_axis) 0 2.5])

axis off

pause(.0001)

end

%%%%% Model Differential Equations %%%%%%

function dx = odefun(t,x)

%passed variables and parameters

x1=x(1);

x2=x(2);

x3=x(3);

x4=x(4);

x5=x(5);

AP_axis=x(6);%position

b_to_g_time=x(7);%blastoderm-to-germband transition time

a=x(8);%starting infliction point of the gradient

v=x(9);%velocity of the wavefront

m=x(10);%starting steepness of the gradient

DyFlag=x(11);%gradient buildup dynamics flag

max_t=x(12);%time of end of simulation

%setting up the gradient G

if(t<b_to_g_time)

u=0;

current_m=m;

else

u=v;

current_m=20*exp(t-b_to_g_time);

if(current_m>100)

current_m=100;

end

end

if(DyFlag==0)

Dy=1;

elseif(DyFlag==1)

Dy=1-(t/max_t-1).^2;

elseif(DyFlag==2)

Dy=1-(2*t/max_t-1).^2;

elseif(DyFlag==3)

Dy=1-(t/max_t).^2;

Dy(Dy<0)=0;

else

error('invalid DyFlag value')

end

G= Dy * 1.5/(1+exp(-current_m*((AP_axis-u*(t-b_to_g_time))-a)));

%gene regulation parameters

w=2.5;%dissociation constant of weak regulatory interactions

s=.2;%dissociation constant of strong regulatory interactions

n=5;%cooperativity constant

lambda=1;%decay rate

%%%%%%%%%%%%%%%%%%%% heat-shock experiment setup %%%%%%%%%%%%%%%%%%

hs=0;

if(t>4 && t<5)

hs=0;%3

end

%%%%%%%%%%%%%%%%%%%%%%%%%%%%%%%%%%%%%%%%%%%%%%%%%%%%%%%%%%%%%%%%%%%

%gene regulation

dx(1)= hs+3*(G/.2)^n/(1+(G/.2)^n)*1/(1+(x2/w)^n)*1/(1+(x3/s)^n)*1/(1+(x4/s)^n)*1/(1+(x5/s)^n)*1/(1+(x1/.8)^n)-lambda*x1;

dx(2)= 3*(G/.6)^n/(1+(G/.6)^n)*1/(1+(x3/w)^n)*1/(1+(x4/s)^n)*1/(1+(x5/s)^n)*1/(1+(x2/.8)^n)-lambda*x2;

dx(3)= 3*(G/1)^n/(1+(G/1)^n)*1/(1+(x4/w)^n)*1/(1+(x5/s)^n)*1/(1+(x3/.8)^n)-lambda*x3;

dx(4)= 3*(G/1.4)^n/(1+(G/1.4)^n)*1/(1+(x5/.4)^n)*1/(1+(x4/.8)^n)-lambda*x4;

dx(5)= 3*(G/1.8)^n/(1+(G/1.8)^n)*1/(1+(x5/.8)^n)-lambda*x5;

%zero change in parameters

dx(7:12)=0;

dx=dx';

**Code C3.** Matlab code for our realization of the French Flag with Timer Gene (FFTG) model

function french_flag_cricuit_timer

clear

clc

close all

%%%%%%%%%%%%%%%%%%%%% PARAMETERS %%%%%%%%%%%%%%%%%%%%%%%%%%%%%%%%%%

%%% For pure short-germ: b_to_g_time=.01; a=.05; m=100;

%%% For pure long germ: b_to_g_time= 10; a=.25; m=20;

%%% For intermediate germ: b_to_g_time= 4; a=.25; m=20;

b_to_g_time=4;%time of blastoderm to germband transition

a=0.25;%starting infliction point of the gradient

m=20;%starting steepness of the gradient

v=.05;%wavefront velocity

%Gradient buildup dynamics

DyFlag=0; %gradient buildup dynamics flag:

%'0' for no buildup or decay dynamics

%'1' for only building up

%'2' for build up then decay

%'3' for only decay

%%%%%%%%%%%%%%%%%%%%%%%%%%%%%%%%%%%%%%%%%%%%%%%%%%%%%%%%%%%%%%%%%55

t=0:.01:10; %time axis

AP_axis=0:.001:.5; % spatial axis

%solve model

spacetime=zeros(6,length(t),length(AP_axis));

for pos=1:length(AP_axis)

initial_conditions=...

[.6 zeros(1,5) AP_axis(pos) b_to_g_time a v m DyFlag max(t)];

[~, x]= ode45(@odefun,t,initial_conditions);

spacetime(:,:,pos)=x(:,1:6)';

end

%plot solution

close all

for nt=1:length(t)

%setting up the gradient G

if(t(nt)<b_to_g_time)

u=0;

current_m=m;

else

u=v;

current_m=m*exp(t(nt)-b_to_g_time);

if(current_m>100)

current_m=100;

end

end

if(DyFlag==0)

Dy=1;

elseif(DyFlag==1)

Dy=1-(t(nt)/max(t)-1).^2;

elseif(DyFlag==2)

Dy=1-(2*t(nt)/max(t)-1).^2;

elseif(DyFlag==3)

Dy=1-(t(nt)/max(t)).^2;

Dy(Dy<0)=0;

else

error('invalid DyFlag value')

end

G= Dy * 1.5./(1+exp(-current_m*((AP_axis-u*(t(nt)-b_to_g_time))-a)));

set(gca, 'ColorOrder',...

[86 128 193; 229 51 50; 79 185 118; 219 152 40; 168 124 78; 0 0 0; 128 128 128]/256,...

'NextPlot', 'replacechildren');

plot(AP_axis,[squeeze(spacetime(:,nt,:))' G'],'LineWidth',5)

axis([0 max(AP_axis) 0 2.5])

axis off

pause(.0001)

end

%%%%% Model Differential Equations %%%%%%

function dx = odefun(t,x)

%passed variables and parameters

x1=x(1);

x2=x(2);

x3=x(3);

x4=x(4);

x5=x(5);

timer=x(6);

AP_axis=x(7);%position

b_to_g_time=x(8);%blastoderm-to-germband transition time

a=x(9);%starting infliction point of the gradient

v=x(10);%velocity of the wavefront

m=x(11);%starting steepness of the gradient

DyFlag=x(12);%gradient buildup dynamics flag

max_t=x(13);%time of end of simulation

%setting up the gradient G

if(t<b_to_g_time)

u=0;

current_m=m;

else

u=v;

current_m=20*exp(t-b_to_g_time);

if(current_m>100)

current_m=100;

end

end

if(DyFlag==0)

Dy=1;

elseif(DyFlag==1)

Dy=1-(t/max_t-1).^2;

elseif(DyFlag==2)

Dy=1-(2*t/max_t-1).^2;

elseif(DyFlag==3)

Dy=1-(t/max_t).^2;

Dy(Dy<0)=0;

else

error('invalid DyFlag value')

end

G= Dy * 1.5/(1+exp(-current_m*((AP_axis-u*(t-b_to_g_time))-a)));

%gene regulation parameters

w=2.5;%dissociation constant of weak regulatory interactions

s=.2;%dissociation constant of strong regulatory interactions

n=5;%cooperativity constant

lambda=1;%decay rate

%%%%%%%%%%%%%%%%%%%% heat-shock experiment setup %%%%%%%%%%%%%%%%%%

hs=0;

if(t>4 && t<5)

hs=0;%3

end

%%%%%%%%%%%%%%%%%%%%%%%%%%%%%%%%%%%%%%%%%%%%%%%%%%%%%%%%%%%%%%%%%%%

%gene regulation

dx(1)= hs+3*(timer/.2)^n/(1+(timer/.2)^n)*1/(1+(x2/w)^n)*1/(1+(x3/s)^n)*1/(1+(x4/s)^n)*1/(1+(x5/s)^n)*1/(1+(x1/.8)^n)-lambda*x1;

dx(2)= 3*(timer/.6)^n/(1+(timer/.6)^n)*1/(1+(x3/w)^n)*1/(1+(x4/s)^n)*1/(1+(x5/s)^n)*1/(1+(x2/.8)^n)-lambda*x2;

dx(3)= 3*(timer/1)^n/(1+(timer/1)^n)*1/(1+(x4/w)^n)*1/(1+(x5/s)^n)*1/(1+(x3/.8)^n)-lambda*x3;

dx(4)= 3*(timer/1.4)^n/(1+(timer/1.4)^n)*1/(1+(x5/.4)^n)*1/(1+(x4/.8)^n)-lambda*x4;

dx(5)= 3*(timer/1.8)^n/(1+(timer/1.8)^n)*1/(1+(x5/.8)^n)-lambda*x5;

dx(6)= 0.15*G;

%zero change in parameters

dx(7:13)=0;

dx=dx';

**Code C4.** Matlab code for ‘Decay Rates Modulation’ realization of the Speed Regulation model

function speed_regulation_decay_rates_cricuit

clear

clc

close all

%%%%%%%%%%%%%%%%%%%%% PARAMETERS %%%%%%%%%%%%%%%%%%%%%%%%%%%%%%%%%%

%%% For pure short-germ: b_to_g_time=.01; a=.05; m=100;

%%% For pure long germ: b_to_g_time= 10; a=.25; m=20;

%%% For intermediate germ: b_to_g_time= 4; a=.25; m=20;

b_to_g_time=4;%time of blastoderm to germband transition

a=.25;%starting infliction point of the gradient

m=20;%starting steepness of the gradient

v=.05;%wavefront velocity

%Gradient buildup dynamics

DyFlag=0; %gradient buildup dynamics flag:

%'0' for no buildup or decay dynamics

%'1' for only building up

%'2' for build up then decay

%'3' for only decay

%%%%%%%%%%%%%%%%%%%%%%%%%%%%%%%%%%%%%%%%%%%%%%%%%%%%%%%%%%%%%%%%%55

t=0:.01:10; %time axis

AP_axis=0:.001:.5; % spatial axis

%solve model

spacetime=zeros(5,length(t),length(AP_axis));

for pos=1:length(AP_axis)

initial_conditions=...

[.6 zeros(1,4) AP_axis(pos) b_to_g_time a v m DyFlag max(t)];

[~, x]= ode45(@odefun,t,initial_conditions);

spacetime(:,:,pos)=x(:,1:5)';

end

%plot solution

close all

for nt=1:length(t)

%setting up the gradient G

if(t(nt)<b_to_g_time)

u=0;

current_m=m;

else

u=v;

current_m=m*exp(t(nt)-b_to_g_time);

if(current_m>100)

current_m=100;

end

end

if(DyFlag==0)

Dy=1;

elseif(DyFlag==1)

Dy=1-(t(nt)/max(t)-1).^2;

elseif(DyFlag==2)

Dy=1-(2*t(nt)/max(t)-1).^2;

elseif(DyFlag==3)

Dy=1-(t(nt)/max(t)).^2;

Dy(Dy<0)=0;

else

error('invalid DyFlag value')

end

G= Dy * 1.5./(1+exp(-current_m*((AP_axis-u*(t(nt)-b_to_g_time))-a)));

set(gca, 'ColorOrder',...

[86 128 193; 229 51 50; 79 185 118; 219 152 40; 168 124 78;128 128 128 ]/256,...

'NextPlot', 'replacechildren');

plot(AP_axis,[squeeze(spacetime(:,nt,:))' G'],'LineWidth',5)

axis([0 max(AP_axis) 0 2.5])

axis off

pause(.0001)

end

%%%%% Model Differential Equations %%%%%%

function dx = odefun(t,x)

%passed variables and parameters

x1=x(1);

x2=x(2);

x3=x(3);

x4=x(4);

x5=x(5);

AP_axis=x(6);%position

b_to_g_time=x(7);%blastoderm-to-germband transition time

a=x(8);%starting infliction point of the gradient

v=x(9);%velocity of the wavefront

m=x(10);%starting steepness of the gradient

DyFlag=x(11);%gradient buildup dynamics flag

max_t=x(12);%time of end of simulation

%setting up the gradient G

if(t<b_to_g_time)

u=0;

current_m=m;

else

u=v;

current_m=20*exp(t-b_to_g_time);

if(current_m>100)

current_m=100;

end

end

if(DyFlag==0)

Dy=1;

elseif(DyFlag==1)

Dy=1-(t/max_t-1).^2;

elseif(DyFlag==2)

Dy=1-(2*t/max_t-1).^2;

elseif(DyFlag==3)

Dy=1-(t/max_t).^2;

Dy(Dy<0)=0;

else

error('invalid DyFlag value')

end

G= Dy * 1.5/(1+exp(-current_m*((AP_axis-u*(t-b_to_g_time))-a)));

%gene regulation parameters

w=2.5;%dissociation constant of weak regulatory interactions

s=.4;%dissociation constant of strong regulatory interactions

n=5;%cooperativity constant

lambda=1;%decay rate

%%%%%%%%%%%%%%%%%%%% heat-shock experiment setup %%%%%%%%%%%%%%%%%%

hs=0;

if(t>4 && t<5)

hs=0;%3

end

%%%%%%%%%%%%%%%%%%%%%%%%%%%%%%%%%%%%%%%%%%%%%%%%%%%%%%%%%%%%%%%%%%%

%gene regulation

dx(1)= hs+G/(1+G)*(1/(1+(x2/s)^n)*1/(1+(x3/s)^n)*1/(1+(x4/s)^n)*1/(1+(x5/s)^n)-lambda*x1);

dx(2)= G/(1+G)*(1/(1+(x1/w)^n)*1/(1+(x3/s)^n)*1/(1+(x4/s)^n)*1/(1+(x5/s)^n)-lambda*x2);

dx(3)= G/(1+G)*(1/(1+(x1/s)^n)*1/(1+(x2/w)^n)*1/(1+(x4/s)^n)*1/(1+(x5/s)^n)-lambda*x3);

dx(4)= G/(1+G)*(1/(1+(x1/s)^n)*1/(1+(x2/s)^n)*1/(1+(x3/w)^n)*1/(1+(x5/s)^n)-lambda*x4);

dx(5)= G/(1+G)*(1/(1+(x1/s)^n)*1/(1+(x2/s)^n)*1/(1+(x3/s)^n)*1/(1+(x4/w)^n)-lambda*x5);

%zero change in parameters

dx(6:12)=0;

dx=dx';

Parameter sets used to generate the simulations documented in the paper

Here we provide the parameter set used in the Matlab codes provided above to generate each of the simulations documented in the paper.

**Video 1**

We used Matlab **Code C2** with the following parameters:

b_to_g_time=11;%time of blastoderm to germband transition

a=.25;%starting infliction point of the gradient

m=20;%starting steepness of the gradient

v=.05;%wavefront velocity

%Gradient buildup dynamics

DyFlag=0; %gradient buildup dynamics flag

%'0' for no buildup or decay dynamics

%'1' for only building up

%'2' for build up then decay

%'3' for only decay

%%%%%%%%%%%%%%%%%%%% heat-shock experiment setup %%%%%%%%%%%%%%%%%%

hs=0;

if(t>4 && t<5)

hs=0;

end

%%%%%%%%%%%%%%%%%%%%%%%%%%%%%%%%%%%%%%%%%%%%%%%%%%%%%%%%%%%%%%%%%%%

**Video 2**

We used Matlab **Code C2** with the following parameters:

b_to_g_time=11;%time of blastoderm to germband transition

a=.25;%starting infliction point of the gradient

m=20;%starting steepness of the gradient

v=.05;%wavefront velocity

%Gradient buildup dynamics

DyFlag=1; %gradient buildup dynamics flag

%'0' for no buildup or decay dynamics

%'1' for only building up

%'2' for build up then decay

%'3' for only decay

%%%%%%%%%%%%%%%%%%%% heat-shock experiment setup %%%%%%%%%%%%%%%%%%

hs=0;

if(t>4 && t<5)

hs=0;

end

%%%%%%%%%%%%%%%%%%%%%%%%%%%%%%%%%%%%%%%%%%%%%%%%%%%%%%%%%%%%%%%%%%%

**Video 3**

We used Matlab **Code C1** with the following parameters:

b_to_g_time=11;%time of blastoderm to germband transition

a=.25;%starting infliction point of the gradient

m=20;%starting steepness of the gradient

v=.05;%wavefront velocity

%Gradient buildup dynamics

DyFlag=0; %gradient buildup dynamics flag

%'0' for no buildup or decay dynamics

%'1' for only building up

%'2' for build up then decay

%'3' for only decay

%%%%%%%%%%%%%%%%%%%% heat-shock experiment setup %%%%%%%%%%%%%%%%%%

hs=0;

if(t>4 && t<5)

hs=0;

end

%%%%%%%%%%%%%%%%%%%%%%%%%%%%%%%%%%%%%%%%%%%%%%%%%%%%%%%%%%%%%%%%%%%

**Video 4**

We used Matlab **Code C1** with the following parameters:

b_to_g_time=11;%time of blastoderm to germband transition

a=.25;%starting infliction point of the gradient

m=20;%starting steepness of the gradient

v=.05;%wavefront velocity

%Gradient buildup dynamics

DyFlag=3; %gradient buildup dynamics flag

%'0' for no buildup or decay dynamics

%'1' for only building up

%'2' for build up then decay

%'3' for only decay

%%%%%%%%%%%%%%%%%%%% heat-shock experiment setup %%%%%%%%%%%%%%%%%%

hs=0;

if(t>4 && t<5)

hs=0;

end

%%%%%%%%%%%%%%%%%%%%%%%%%%%%%%%%%%%%%%%%%%%%%%%%%%%%%%%%%%%%%%%%%%%

**Video 5**

We used Matlab **Code C1** with the following parameters:

b_to_g_time=11;%time of blastoderm to germband transition

a=.25;%starting infliction point of the gradient

m=20;%starting steepness of the gradient

v=.05;%wavefront velocity

%Gradient buildup dynamics

DyFlag=2; %gradient buildup dynamics flag

%'0' for no buildup or decay dynamics

%'1' for only building up

%'2' for build up then decay

%'3' for only decay

%%%%%%%%%%%%%%%%%%%% heat-shock experiment setup %%%%%%%%%%%%%%%%%%

hs=0;

if(t>4 && t<5)

hs=0;

end

%%%%%%%%%%%%%%%%%%%%%%%%%%%%%%%%%%%%%%%%%%%%%%%%%%%%%%%%%%%%%%%%%%%

**Video 6 (A)**

We used Matlab **Code C3** with the following parameters:

b_to_g_time=0.01;%time of blastoderm to germband transition

a=.05;%starting infliction point of the gradient

m=100;%starting steepness of the gradient

v=.05;%wavefront velocity

%Gradient buildup dynamics

DyFlag=0; %gradient buildup dynamics flag

%'0' for no buildup or decay dynamics

%'1' for only building up

%'2' for build up then decay

%'3' for only decay

%%%%%%%%%%%%%%%%%%%% heat-shock experiment setup %%%%%%%%%%%%%%%%%%

hs=0;

if(t>4 && t<5)

hs=0;

end

%%%%%%%%%%%%%%%%%%%%%%%%%%%%%%%%%%%%%%%%%%%%%%%%%%%%%%%%%%%%%%%%%%%

**Video 6 (B)**

We used Matlab **Code C3** with the following parameters:

b_to_g_time=4;%time of blastoderm to germband transition

a=.25;%starting infliction point of the gradient

m=20;%starting steepness of the gradient

v=.05;%wavefront velocity

%Gradient buildup dynamics

DyFlag=0; %gradient buildup dynamics flag

%'0' for no buildup or decay dynamics

%'1' for only building up

%'2' for build up then decay

%'3' for only decay

%%%%%%%%%%%%%%%%%%%% heat-shock experiment setup %%%%%%%%%%%%%%%%%%

hs=0;

if(t>4 && t<5)

hs=0;

end

%%%%%%%%%%%%%%%%%%%%%%%%%%%%%%%%%%%%%%%%%%%%%%%%%%%%%%%%%%%%%%%%%%%

**Video 6 (C)**

We used Matlab **Code C3** with the following parameters:

b_to_g_time=11;%time of blastoderm to germband transition

a=.25;%starting infliction point of the gradient

m=20;%starting steepness of the gradient

v=.05;%wavefront velocity

%Gradient buildup dynamics

DyFlag=0; %gradient buildup dynamics flag

%'0' for no buildup or decay dynamics

%'1' for only building up

%'2' for build up then decay

%'3' for only decay

%%%%%%%%%%%%%%%%%%%% heat-shock experiment setup %%%%%%%%%%%%%%%%%%

hs=0;

if(t>4 && t<5)

hs=0;

end

%%%%%%%%%%%%%%%%%%%%%%%%%%%%%%%%%%%%%%%%%%%%%%%%%%%%%%%%%%%%%%%%%%%

**Video 7 (A)**

We used Matlab **Code C1** with the following parameters:

b_to_g_time=0.01;%time of blastoderm to germband transition

a=.05;%starting infliction point of the gradient

m=100;%starting steepness of the gradient

v=.05;%wavefront velocity

%Gradient buildup dynamics

DyFlag=0; %gradient buildup dynamics flag

%'0' for no buildup or decay dynamics

%'1' for only building up

%'2' for build up then decay

%'3' for only decay

%%%%%%%%%%%%%%%%%%%% heat-shock experiment setup %%%%%%%%%%%%%%%%%%

hs=0;

if(t>4 && t<5)

hs=0;

end

%%%%%%%%%%%%%%%%%%%%%%%%%%%%%%%%%%%%%%%%%%%%%%%%%%%%%%%%%%%%%%%%%%%

**Video 7 (B)**

We used Matlab **Code C1** with the following parameters:

b_to_g_time=2;%time of blastoderm to germband transition

a=.25;%starting infliction point of the gradient

m=20;%starting steepness of the gradient

v=.05;%wavefront velocity

%Gradient buildup dynamics

DyFlag=0; %gradient buildup dynamics flag

%'0' for no buildup or decay dynamics

%'1' for only building up

%'2' for build up then decay

%'3' for only decay

%%%%%%%%%%%%%%%%%%%% heat-shock experiment setup %%%%%%%%%%%%%%%%%%

hs=0;

if(t>4 && t<5)

hs=0;

end

%%%%%%%%%%%%%%%%%%%%%%%%%%%%%%%%%%%%%%%%%%%%%%%%%%%%%%%%%%%%%%%%%%%

**Video 7 (C)**

We used Matlab **Code C1** with the following parameters:

b_to_g_time=11;%time of blastoderm to germband transition

a=.25;%starting infliction point of the gradient

m=20;%starting steepness of the gradient

v=.05;%wavefront velocity

%Gradient buildup dynamics

DyFlag=0; %gradient buildup dynamics flag

%'0' for no buildup or decay dynamics

%'1' for only building up

%'2' for build up then decay

%'3' for only decay

%%%%%%%%%%%%%%%%%%%% heat-shock experiment setup %%%%%%%%%%%%%%%%%%

hs=0;

if(t>4 && t<5)

hs=0;

end

%%%%%%%%%%%%%%%%%%%%%%%%%%%%%%%%%%%%%%%%%%%%%%%%%%%%%%%%%%%%%%%%%%%

**Video 8**

We used Matlab **Code C1** with the following parameters:

b_to_g_time=2;%time of blastoderm to germband transition

a=.25;%starting infliction point of the gradient

m=20;%starting steepness of the gradient

v=.05;%wavefront velocity

%Gradient buildup dynamics

DyFlag=0; %gradient buildup dynamics flag

%'0' for no buildup or decay dynamics

%'1' for only building up

%'2' for build up then decay

%'3' for only decay

%%%%%%%%%%%%%%%%%%%% heat-shock experiment setup %%%%%%%%%%%%%%%%%%

hs=0;

if(t>3 && t<4)

hs=3;

end

%%%%%%%%%%%%%%%%%%%%%%%%%%%%%%%%%%%%%%%%%%%%%%%%%%%%%%%%%%%%%%%%%%%

**Video 9**

We used Matlab **Code C3** with the following parameters:

b_to_g_time=2;%time of blastoderm to germband transition

a=.25;%starting infliction point of the gradient

m=20;%starting steepness of the gradient

v=.05;%wavefront velocity

%Gradient buildup dynamics

DyFlag=0; %gradient buildup dynamics flag

%'0' for no buildup or decay dynamics

%'1' for only building up

%'2' for build up then decay

%'3' for only decay

%%%%%%%%%%%%%%%%%%%% heat-shock experiment setup %%%%%%%%%%%%%%%%%%

hs=0;

if(t>5 && t<6)

hs=3;

end

%%%%%%%%%%%%%%%%%%%%%%%%%%%%%%%%%%%%%%%%%%%%%%%%%%%%%%%%%%%%%%%%%%%

**Video 10**

We used Matlab **Code C4** with the following parameters:

b_to_g_time=4;%time of blastoderm to germband transition

a=.25;%starting infliction point of the gradient

m=20;%starting steepness of the gradient

v=.05;%wavefront velocity

%Gradient buildup dynamics

DyFlag=0; %gradient buildup dynamics flag

%'0' for no buildup or decay dynamics

%'1' for only building up

%'2' for build up then decay

%'3' for only decay

%%%%%%%%%%%%%%%%%%%% heat-shock experiment setup %%%%%%%%%%%%%%%%%%

hs=0;

if(t>5 && t<6)

hs=0;

end

%%%%%%%%%%%%%%%%%%%%%%%%%%%%%%%%%%%%%%%%%%%%%%%%%%%%%%%%%%%%%%%%%%%

**Video 11**

We used Matlab **Code C4** with the following parameters:

b_to_g_time=4;%time of blastoderm to germband transition

a=.25;%starting infliction point of the gradient

m=20;%starting steepness of the gradient

v=.05;%wavefront velocity

%Gradient buildup dynamics

DyFlag=0; %gradient buildup dynamics flag

%'0' for no buildup or decay dynamics

%'1' for only building up

%'2' for build up then decay

%'3' for only decay

%%%%%%%%%%%%%%%%%%%% heat-shock experiment setup %%%%%%%%%%%%%%%%%%

hs=0;

if(t>5 && t<6)

hs=3;

end

%%%%%%%%%%%%%%%%%%%%%%%%%%%%%%%%%%%%%%%%%%%%%%%%%%%%%%%%%%%%%%%%%%%

**Video 12 (A)**

We used Matlab **Code C1** with the following parameters:

b_to_g_time=11;%time of blastoderm to germband transition

a=.25;%starting infliction point of the gradient

m=20;%starting steepness of the gradient

v=.05;%wavefront velocity

%Gradient buildup dynamics

DyFlag=0; %gradient buildup dynamics flag

%'0' for no buildup or decay dynamics

%'1' for only building up

%'2' for build up then decay

%'3' for only decay

%%%%%%%%%%%%%%%%%%%% heat-shock experiment setup %%%%%%%%%%%%%%%%%%

hs=0;

if(t>4 && t<5)

hs=0;

end

%%%%%%%%%%%%%%%%%%%%%%%%%%%%%%%%%%%%%%%%%%%%%%%%%%%%%%%%%%%%%%%%%%%

**Video 12 (B)**

We used Matlab **Code C1** with the following parameters:

b_to_g_time=11;%time of blastoderm to germband transition

a=.25;%starting infliction point of the gradient

m=20;%starting steepness of the gradient

v=.05;%wavefront velocity

%Gradient buildup dynamics

DyFlag=0; %gradient buildup dynamics flag

%'0' for no buildup or decay dynamics

%'1' for only building up

%'2' for build up then decay

%'3' for only decay

%%%%%%%%%%%%%%%%%%%% heat-shock experiment setup %%%%%%%%%%%%%%%%%%

hs=0;

if(t>4 && t<5)

hs=3;

end

%%%%%%%%%%%%%%%%%%%%%%%%%%%%%%%%%%%%%%%%%%%%%%%%%%%%%%%%%%%%%%%%%%%
